# Coexistence of high-risk HPV infection and FANCC heterozygous variation in early young esophageal squamous cell carcinoma: a case report and literature review

**DOI:** 10.1186/s12876-026-05162-0

**Published:** 2026-08-14

**Authors:** Lei Wu, Jiyong Jing, Chenjing Zhang, Wensheng Pan

**Affiliations:** 1 Wenzhou Medical University, Wenzhou, Zhejiang China; 2 Zhejiang Provincial People’s Hospital, Hangzhou, Zhejiang China

**Keywords:** Esophageal cancer, Young patients, Endoscopic submucosal dissection (ESD), HPV infection, DNA repair genes, Early diagnosis

## Abstract

**Background:**

Esophageal cancer poses a major health challenge in China, with current screening targeting individuals over 40. However, rising incidence in younger populations, often with atypical symptoms, leads to delayed diagnosis. Investigating the underlying causes in this group is urgently needed.

**Case introduction:**

We report a case of a 28-year-old male with early esophageal squamous cell carcinoma presenting with nonspecific abdominal distension and diarrhea. Lesions were identified using magnifying endoscopy with narrowband imaging and treated via endoscopic submucosal dissection (ESD). Postoperative pathology confirmed carcinoma in situ. Molecular analysis revealed high-risk HPV 16/18 infection and whole exome sequencing showed DNA repair gene alterations, including heterozygous FANCC variants. A review of 12 PubMed cases of patients under 30 highlighted varied and atypical presentations, often diagnosed at advanced stages with poor prognosis.

**Conclusion:**

This case highlights the importance of clinical vigilance for young individuals with persistent symptoms and risk factors, serving as a potential complement to current age-based screening guidelines. The RNAscope finding (score 3) highly suggests HPV-driven oncogenesis based on established criteria in HPV-associated cancers. A germline heterozygous FANCC VUS was also identified, but no mechanistic conclusion can be drawn from this finding without functional validation. The coexistence of these two findings provides a hypothesis-generating clue for future research.

## Introduction

Esophageal cancer is the eighth most prevalent malignant tumor globally, with China experiencing a particularly significant burden, as it reports the highest number of cases worldwide [1]. Current screening guidelines typically recommend initiating screening at age 40 or older. In younger individuals, esophageal cancer is frequently misdiagnosed as gastroesophageal reflux disease or functional disorders due to atypical symptoms, leading to the majority of patients receiving a diagnosis at an advanced stage [2]. Endoscopic techniques are essential for early detection and treatment, particularly endoscopic submucosal dissection (ESD), which has emerged as a radical minimally invasive treatment for early-stage mucosal cancer. This study offers a new theoretical perspective on the etiology and mechanisms of such diseases through an in-depth molecular pathological analysis of a case of early esophageal cancer in a young patient.

## Case report

The patient is a 28-year-old male who presented to another hospital with abdominal distension, diarrhea, and nausea. Gastroscopy revealed a type IIa lesion in the esophageal mucosa, raising suspicion for early esophageal cancer. He subsequently transferred to our hospital for further diagnosis and treatment. The patient had a history of gastroesophageal reflux and Helicobacter pylori infection.

Clinical characteristics: The patient denied any history of tobacco smoking, alcohol consumption, or use of other recreational substances. His body mass index (BMI) was 26.3 kg/m². He reported no habitual consumption of very hot beverages, pickled vegetables, or processed meats. There was no documented occupational exposure to known carcinogens (e.g., chemical solvents, heavy metals, dusts). A three-generation family history was negative for esophageal cancer or any other malignancy.

### Diagnostic evaluation

**Fig. 1:**
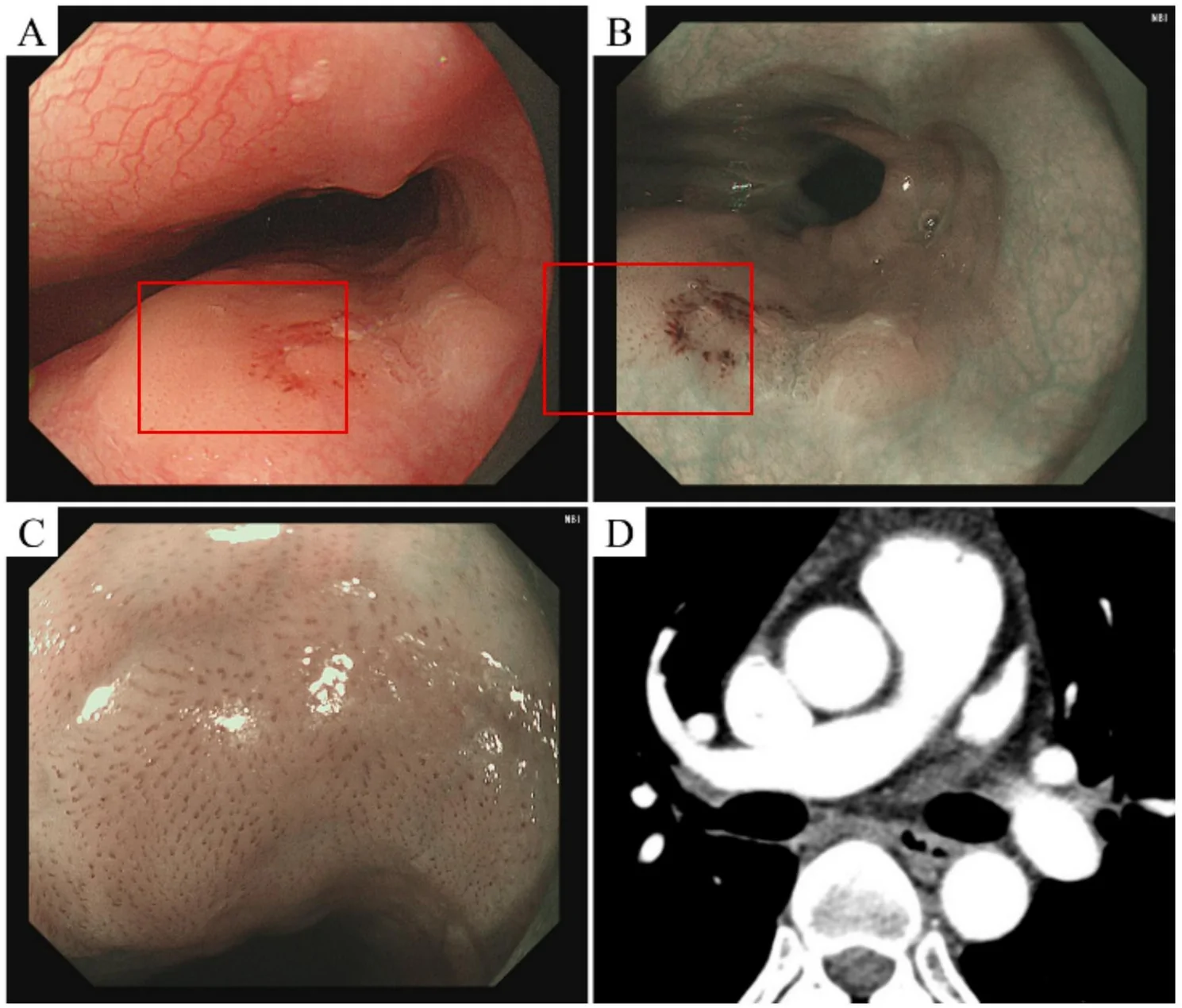
Endoscopic and CT findings of the esophageal lesion. **A **White-light endoscopy shows a type IIa lesion; **B** Narrow-band imaging (NBI) reveals a brownish area; **C** Magnifying endoscopy shows dilated and tortuous IPCLs (Type B1); **D** Contrast-enhanced CT shows no obvious wall thickening, stenosis, or lymph node metastasis

Magnifying endoscopy combined with narrow-band imaging (NBI) was conducted. This examination revealed that the mucosa located 33 to 35 cm from the incisors exhibited a brown vascular area and a type IIa lesion, which encompassed approximately 1/2 to 2/3 of the esophageal circumference, strongly suggesting early esophageal cancer. Magnifying endoscopy with NBI revealed dilated and tortuous intrapapillary capillary loops (IPCLs) classified as Type B1 according to the Japan Esophageal Society classification, indicating a lesion confined to the epithelium or lamina propria mucosa, consistent with an endoscopic resectable lesion. An enhanced CT examination indicated that the esophageal lumen remained unobstructed, with no discernible enhancement foci, stenosis, or signs of lymph node metastasis (Fig. 1).

### Treatment

**Fig. 2:**
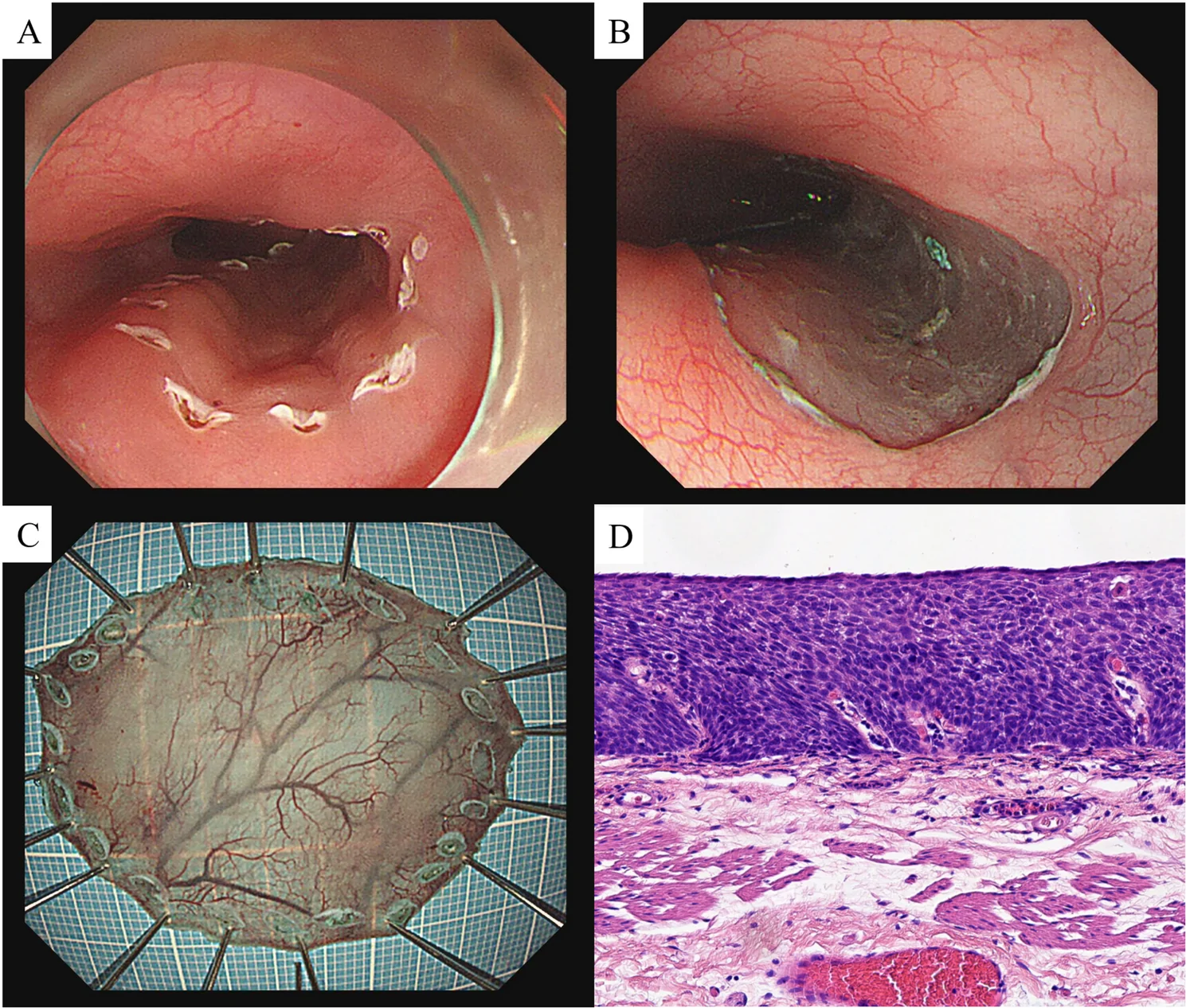
Endoscopic resection and histopathological examination. **A** APC demarcation of the lesion margin; **B** ESD procedure in process; **C** Gross appearance of the resected specimen (55×35 mm); **D **H&E staining confirming squamous cell carcinoma in situ

Following the assessment confirming the absence of surgical contraindications, ESD was performed. The procedure proceeded without incident, resulting in a complete resection. Postoperative evaluation revealed no complications, including bleeding or perforation. The pathological diagnosis indicated carcinoma in situ (pTis, N0, M0, Stage 0). The tumor was confined to the epithelium, with no invasion into the lamina propria or muscularis mucosa. Lymphovascular invasion was absent. Both lateral and vertical resection margins were negative for tumor cells (R0 resection) (Fig. 2).

**Fig. 3:**
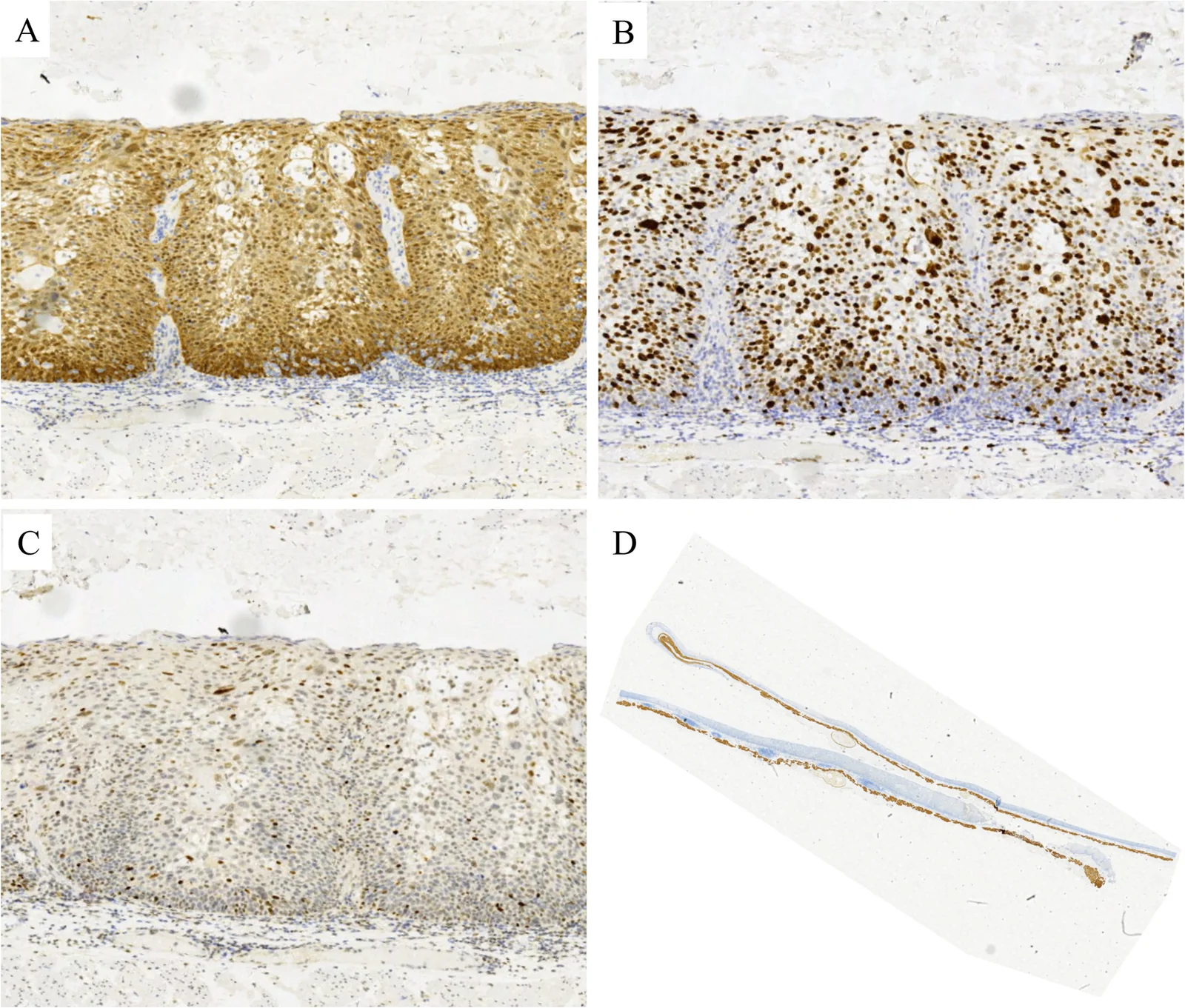
Immunohistochemistry showed: (**A**) strong diffuse p16 positivity, (**B**) high Ki-67 proliferative index (80%), (**C**) wild-type p53 expression pattern, and (**D**) the muscularis mucosa was intact

Immunohistochemical analysis demonstrated positive expression of P40, P63, CK5/6, and pan-cytokeratin (Pan-CK) in the tumor cells, indicating squamous differentiation. The lack of CK7 expression suggests the absence of glandular differentiation. The expression pattern of wild-type p53 was noted, accompanied by strong diffuse positivity for p16. The proliferation marker Ki-67 exhibited a high proliferation index of 80%. The mucosal muscular layer remained intact (Fig. 3).

Postoperatively, RNAscope technology was employed to perform in situ hybridization for the detection of high-risk HPV subtypes (16/18) E6/E7 mRNA in the tumor tissue samples. The results indicated focal HPV 16/18 signals with an ACD score of 3 points (moderate-to-strong positivity), defined as 10–15 or more punctate signals per nucleus or small clusters under 40× magnification, reflecting highly active viral transcription.

Whole-exome sequencing (WES) was performed on genomic DNA extracted from the patient’s peripheral blood. The average sequencing depth was 244.38×, with > 99.5% of targeted regions covered at ≥ 20×. Variants were annotated using the Ensembl Variant Effect Predictor and filtered against dbSNP, 1000 Genomes, gnomAD (v2.1), and the Genome Aggregation Database. Pathogenicity was classified according to ACMG/AMP 2015 guidelines (Table 1).

**Table 1:** Clinically relevant variations detected by whole exome sequencing

Gene	Zygosity	Pathogenicity Classification	Associated Disease	Inheritance Pattern
SUOX	HET	P	Sulfite oxidase deficiency	AR
FANCC	HET	VUS	Fanconi anemia complementation group C	AR
DNHD1	HET	LP	Spermatogenic failure 65	AR

*P* Pathogenic, *LP* Likely Pathogenic, *VUS* Variants of Uncertain Significance, *HET *Heterozygous variant, *AR* Autosomal recessive

### Follow-up

The patient underwent regular endoscopic follow-up after the ESD procedure performed in March 2024. Surveillance endoscopies were conducted at 2, 5, 11, and 18 months post-procedure. As of the most recent follow-up at 18 months (September 2025), no local recurrence or metachronous lesion has been observed. Continued annual surveillance is planned. The patient was advised to modify lifestyle habits, including smoking cessation, limiting alcohol consumption, and optimizing diet, to mitigate the risk of recurrence.

### Literature summary and review

To provide clinical context for early-onset esophageal cancer, we conducted a narrative review of the PubMed literature rather than a systematic review. The search was performed using the terms “esophageal cancer,” “young adult,” “early-onset,” and “case report” for English-language publications available up to December 31, 2025. No formal quality assessment or meta-analysis was performed. Given the inherent publication bias and heterogeneity of case reports, the following summary should be interpreted as illustrative rather than epidemiologically representative.

We identified and summarized a total of 12 young patients, aged between 17 and 30 years (10 males and 2 females), diagnosed with esophageal cancer. The predominant clinical manifestations included progressive dysphagia (7 cases, 58.3%), weight loss (7 cases, 58.3%), and chest pain or reflux symptoms (4 cases, 33.3%). Other atypical symptoms encompassed hematemesis, pericarditis, headache, and hemiplegia. Regarding pathological types, esophageal squamous cell carcinoma was the most prevalent, accounting for 7 cases (58.3%), followed by adenocarcinoma in 3 cases (25.0%). Additionally, rare types such as sarcomatoid carcinoma and rhabdoid tumor were noted. At diagnosis, tumors were generally at an advanced stage, with 75.0% of patients (9/12) presenting at Stage III or IV, most of whom had lymph node or distant metastasis. Treatment options included esophagectomy, neoadjuvant chemoradiotherapy, endoscopic intervention, and palliative care. Among the cases with reported outcomes, over half (6/11, 54.5%) experienced disease progression, metastasis, or death within several months post-diagnosis, while only a few early-stage cases achieved long-term survival through radical surgery or endoscopic treatment (Table 2).

**Table 2:** List of young patients with esophageal cancer reported in the literature

Age/Sex	Clinical Presentation	Pathology	TNM Stage	Treatment	Outcome
17 /M [3]	Progressive dysphagia, weight loss (14 kg)	Squamous cell carcinoma	cT4aN0M0 (Stage III)	Neoadjuvant chemoradiation→Thoracoscopy-assisted esophagectomy	Successfully discharged, under follow-up
20s /M [4]	Chest pain, pericarditis, dysphagia, weight loss	Squamous cell carcinoma	cT4aNXM1 (Stage IV)	Pericarditis management → Palliative chemotherapy	Died a few months later
23/ M [5]	Postprandial chest pain, acid reflux, hemoptysis	Sarcomatoid carcinoma (suspected)	cT_N_M1 (Stage IV)	Surgery avoided after multidisciplinary consultation	Alive at 3-year follow-up
24/M [6]	Progressive dysphagia, retrosternal pain, weight loss (15 kg)	Rhabdoid tumor	cT3N1M0 (Stage III)	Minimally invasive Ivor Lewis esophagectomy	Liver/bone metastases at 2 months, died at 3 months
24/M [7]	Dysphagia, vomiting, weight loss	Adenocarcinoma	pT3N1M0 (Stage III)	Ivor Lewis esophagectomy → Stent → Surgery for fistula	Died 6 months post-op from peritoneal carcinomatosis (M1)
25/F [8]	Dysphagia, weight lo ss	Squamous cell carcinoma	cT1bN0M0 (Stage I)	Neoadjuvant chemoradiation → VATS esophagectomy	No recurrence at 6 months, complications e.g., hoarseness
28/M [9]	Headache, hemiplegia	Adenocarcinoma	cTXN1M1 (Stage IV)	Craniotomy + Whole-brain radiotherapy	Transferred for further treatment, poor prognosis
29/M [10]	Hematemesis, dysphagia, weight loss	Squamous cell carcinoma	cT1bN0M0 (Stage I)	Endoscopic biopsy, referred to oncology	Not reported
28/M [11]	Depression, pyrosis, dysphagia	Adenocarcinoma	cT3N1M1 (Stage IV)	Exploratory surgery, unresectable	Died within 6 months
30 F [12]	Feeling of stuffiness when swallowing	Squamous cell carcinoma	pT3N1M0 (Stage III)	Esophagectomy	Died 8 months post-op from bone metastases (M1)
21 F [12]	Cervical lymphadenopathy	Squamous cell carcinoma	cT1N2M0 (Stage III)	Chemoradiation + Endoscopic ablation	Died 5 years after diagnosis
26 F [12]	Swallowing discomfort	Squamous cell carcinoma	pT1bN0M0 (Stage I)	Esophagectomy	Alive without recurrence at 5 years 10 months

## Discussion

This study presents a case of early esophageal squamous cell carcinoma in a 28-year-old male, whose clinical manifestations were limited to non-specific symptoms, including abdominal distension and diarrhea. The use of magnifying endoscopy with narrow-band imaging allowed precise lesion localization, and en bloc resection by endoscopic submucosal dissection achieved a minimally invasive radical cure. Postoperative pathology confirmed carcinoma in situ. This diagnostic and therapeutic pathway underscores the insidious nature of esophageal cancer in younger individuals and highlights the importance of early intervention. The molecular findings—specifically, the detection of transcriptionally active high-risk HPV and a germline heterozygous variant of uncertain significance (VUS) in FANCC—offer a hypothesis-generating perspective for future research into early-onset esophageal cancer. We selected this case for detailed reporting due to its clinical warning significance and its potential value in generating mechanistic hypotheses.

### Thoughts on screening timing: a strategy based on risk stratification

Current domestic and international guidelines generally recommend the initiation of endoscopic screening at age 40 or older. This age threshold is primarily informed by population epidemiological data and aims to balance the costs and benefits of screening. However, findings from this case, along with the accompanying literature review (Table 2), reveal that the predominant clinical manifestations of esophageal cancer in younger individuals are dysphagia (58.3%) and weight loss (58.3%). Notably, approximately one-third of cases present with atypical symptoms, such as chest pain, reflux, and even pericarditis or neurological symptoms, which may lead to delayed diagnosis. A substantial proportion of cases are identified at an advanced stage, with 75.0% of the reviewed cases classified as stage III/IV, resulting in a markedly poor prognosis.

Given that current screening guidelines primarily target the general population, it is worth considering whether more individualized approaches might be beneficial for younger individuals who present with certain clinical features. Based on our case and the limited literature, several factors may warrant clinical attention, including: (1) certain lifestyle habits, such as heavy alcohol consumption, tobacco smoking, or frequent intake of very hot foods and beverages; (2) A family history of digestive tract malignancies among first-degree relatives, or residence in high-incidence regions for esophageal cancer; (3) Carcinogenic virus infection is characterized by a history of persistent high-risk human papillomavirus (HPV) infection [13]; (4) Persistent unexplained upper gastrointestinal symptoms (e.g., dysphagia, weight loss, or symptoms refractory to empirical treatment for > 4–8 weeks) [14].Other conditions such as achalasia or a history of head and neck squamous cell carcinoma may also raise suspicion [7].

Nevertheless, it must be strongly emphasized that these factors are largely hypothetical when applied to young populations, derived from anecdotal case observations rather than robust epidemiological or prospective data. They should not be misinterpreted as formal screening indications or as evidence supporting a change to existing age-based guidelines. Rather, they may serve as contextual clues to increase clinical vigilance when evaluating young patients with persistent symptoms. A lower threshold for endoscopic referral could be considered on a case-by-case basis, but this remains a clinical judgment rather than an evidence-based recommendation. Ultimately, this risk-stratified awareness may serve as a thoughtful complement to the current framework, but prospective cohort studies are urgently needed to validate whether such an approach improves outcomes.

### On the potential role of hpv infection

Applying established RNAscope criteria to the esophagus remains challenging due to ongoing controversy regarding the role of HPV in esophageal squamous cell carcinogenesis. Reported detection rates vary widely (0% to > 50%), and mechanistic evidence remains conflicting [15, 16]. Nevertheless, in this specific case, the presence of a RNAscope score 3 for E6/E7 mRNA, together with diffuse p16 expression, is highly suggestive of HPV-driven oncogenesis. This interpretation is consistent with the biological paradigm established in other HPV-associated cancers [17]. Definitive proof would ideally require additional evidence such as viral integration site analysis or functional assays, but the current findings provide strong supportive evidence.

### On the FANCC VUS — a hypothesis-generating finding, not evidence of causality

The FANCC variant identified in this patient is a heterozygous in‑frame deletion classified as a VUS .Several points must be emphasized to avoid overinterpretation.

First, this variant alone does not confer a diagnosis of Fanconi anemia (FA). FA is a classic chromosomal instability disorder caused by biallelic (or, in the case of FANCB, hemizygous) pathogenic variants in FA pathway genes, leading to impaired DNA interstrand crosslink repair [8]. Heterozygous carriers of FA gene variants generally do not exhibit the characteristic bone marrow failure, congenital anomalies, or cancer susceptibility seen in fully penetrant FA. Therefore, the well‑established association between FA and ESCC [3] cannot be directly extrapolated to this patient.

Second, the current literature provides no functional evidence that a heterozygous FANCC VUS alone impairs DNA repair capacity or increases cancer risk, either in the esophagus or in other organs. Large population-based studies have not identified heterozygous FANCC VUS as a risk factor for ESCC or other solid tumors. Thus, the FANCC finding in this case should be viewed as purely hypothesis‑generating—a coincidental observation that merits further investigation, not as evidence of a pathogenic or synergistic mechanism.

Third, the strength of evidence differs substantially between the two molecular findings in this case. For HPV, the RNAscope score of 3 provides clinically significant evidence of active viral transcription, strongly suggesting a driver role. In contrast, the FANCC VUS remains a variant with no established functional defect. The coexistence of HPV and a FANCC VUS should not be overinterpreted as evidence of “viral‑genetic synergy” without functional validation in appropriate models (e.g., cell lines or animal studies). At present, this coexistence represents only an observational association, not a mechanistic interaction.

As noted in the Genetic Analysis section, the patient also carried a pathogenic SUOX variant and a likely pathogenic DNHD1 variant. Both are associated with autosomal recessive conditions not linked to cancer susceptibility [18, 19]. These variants are likely incidental findings unrelated to esophageal carcinogenesis and are not discussed further.

The clinical utility and ethical implications of returning VUS results to young individuals, especially in the absence of a clear cancer syndrome, must be carefully considered in any future risk assessment framework. Incidental findings such as VUS may cause unnecessary anxiety without providing actionable clinical guidance.

This study has several limitations. First, as a case report, its findings require validation in larger prospective cohorts or case-control studies. Second, while the RNAscope finding (score 3) highly suggests HPV-driven oncogenesis, the role of HPV in ESCC remains controversial, and confirmation in esophageal cancer specifically would benefit from additional evidence such as viral integration analysis. Third, the FANCC variant is classified as a VUS; although it has been reported in a gastroesophageal junction cancer case, its precise pathogenicity requires confirmation through family segregation studies or functional assays. Fourth, parental samples were not available for inheritance pattern analysis. Fifth, the narrative literature review was not systematic and may be subject to selection bias.

## Conclusion

This case reports early-onset ESCC in a 28-year-old male successfully treated by ESD. RNAscope (score 3) highly suggests HPV-driven oncogenesis based on established criteria.A germline heterozygous FANCC VUS was also identified, but this variant does not diagnose Fanconi anemia, and no functional evidence supports a causal or synergistic role. This finding is hypothesis-generating only.Clinically, this case does not support changing current age-based screening guidelines .For young individuals with risk factors or persistent symptoms, endoscopic evaluation may be considered as a complement to the existing framework.

## Data Availability

The datasets used and/or analysed during the current study are available from the corresponding author on reasonable request.
